# The demand control model and circadian saliva cortisol variations in a Swedish population based sample (The PART study)

**DOI:** 10.1186/1471-2458-6-288

**Published:** 2006-11-27

**Authors:** Magnus Alderling, Töres Theorell, Bartolomé de la Torre, Ingvar Lundberg

**Affiliations:** 1 Division of Occupational Medicine, Department of Public Health Sciences, Karolinska Institutet, Stockholm, Sweden; 2 National Institute for Psychosocial Factors and Health, Karolinska Institutet, Stockholm, Sweden; 3 National Institute for Working Life, Stockholm, Sweden

## Abstract

**Background:**

Previous studies of the relationship between job strain and blood or saliva cortisol levels have been small and based on selected occupational groups. Our aim was to examine the association between job strain and saliva cortisol levels in a population-based study in which a number of potential confounders could be adjusted for.

**Methods:**

The material derives from a population-based study in Stockholm on mental health and its potential determinants. Two data collections were performed three years apart with more than 8500 subjects responding to a questionnaire in both waves. In this paper our analyses are based on 529 individuals who held a job, participated in both waves as well as in an interview linked to the second wave. They gave saliva samples at awakening, half an hour later, at lunchtime and before going to bed on a weekday in close connection with the interview. Job control and job demands were assessed from the questionnaire in the second wave. Mixed models were used to analyse the association between the demand control model and saliva cortisol.

**Results:**

Women in low strain jobs (high control and low demands) had significantly lower cortisol levels half an hour after awakening than women in high strain (low control and high demands), active (high control and high demands) or passive jobs (low control and low demands). There were no significant differences between the groups during other parts of the day and furthermore there was no difference between the job strain, active and passive groups. For men, no differences were found between demand control groups.

**Conclusion:**

This population-based study, on a relatively large sample, weakly support the hypothesis that the demand control model is associated with saliva cortisol concentrations.

## Background

Serum cortisol is a widely accepted indicator of energy mobilisation and hence a useful indicator of stress [1]. It has been discovered by Kirschbaum and Hellhammer and confirmed by other groups that variations in saliva concentration reliably reflect variations in the serum concentration of free cortisol [2,3]. Several studies of saliva cortisol variations during the normal round of life and in relation to stressful experiences in several normal groups have been published. However, few studies of representative working population groups have been studied. In the present study the relationship between one of the more widely used theoretical job stress models, the demand control model, and circadian variations in saliva cortisol has been explored.

Previous studies have shown that there are elevated levels of saliva cortisol during the early morning hours in subjects with high demands and low decision latitude (control). The findings have not been consistent however [4-8]. One of the reasons for this could be that subjects with marked disturbances of the capacity to regulate cortisol excretion could distort such a relationship. For instance, studies of serum cortisol variations in patients with severe long lasting psychiatric depression have shown that these subjects are frequently unable to lower their cortisol concentration in the evening [9]. This has also corresponded to inability in a large proportion of these subjects to lower serum cortisol during the dexamethasone test [9]. Subjects with this inability to lower cortisol ("high curves") are less likely than others to respond in the normal way to stressors in everyday life. Similarly it has been discovered that subjects with pronounced symptoms of exhaustion such as the chronic fatigue syndrome are unable to raise their cortisol level in challenging situations and they also show very small circadian variation ("low flat curves") [10,11]. These physiological processes may be behind some of the contradictory findings. It could be that the proportion of subjects who have been exposed for a very long time to adverse conditions – in ways which may influence the regulatory capacity – has been different in different study samples. In the present study a representative sample of the actively working population in Stockholm was studied. This means that serious depression and exhaustion making active work impossible could not disturb our findings. Another reason behind inconsistent findings may be differences in the prevalence of potential confounders – such as smoking habits, alcohol consumption, body mass index and medication – between study samples. In the present study it was possible to explore the importance of such factors to possible relationships between the demand control model and saliva cortisol concentration medication.

## Methods

### Study group

The study was performed within the PART-project. An initial data collection took place in 1998–2000 when a questionnaire was sent to 19742 individuals randomly selected from the population in Stockholm County, 20–64 years of age. The PART Study was approved by the Ethical Committee at Karolinska Institutet as being in accordance with ethical standards (Dnr 96–260 and 01–218). The participants in this project have given written consent after having read a detailed written description of the research. The questionnaire contained questions on potential risk indicators for psychiatric disorder as well as scales to measure well-being, depression and other symptomatology. The response rate was 53%, 58% among women and 48% among men, i.e. 10441 responded. A register-based non-response analysis showed that risk indicators for psychiatric disorders as well as the disorders themselves were more common among non-participants than among participants. However, the relationships between the risk indicators and psychiatric disorders were remarkably similar among participants and non-participants [12]. To the respondents we sent a new questionnaire in 2001–2003 with basically the same questions as in the first questionnaire. Now 84% responded which corresponded to 8613 individuals. Of these, 881 were interviewed regarding psychiatric symptoms and living conditions, 444 were selected because of low well-being defined as ≤ 10 points in the WHO (Ten) Well-being index and 437 were selected because of high well-being defined as >10 points in the same index [13]. Before the interview, which for 80 % of the subjects took place within eight weeks after the questionnaire had been returned, the Well-being index was again filled in.

Participants in the psychiatric interview were asked to deliver saliva samples at awakening (first), half an hour later (second), at lunch (third) and immediately before going to bed (fourth) during a Tuesday, Wednesday, Thursday or Friday in as close connection to the interview as possible. Standard swabs were used. The participants were asked to keep the swabs in their mouth until they were totally swamped by saliva and then to place them in tubes, which were mailed to the laboratory. At the laboratory all samples were centrifuged and frozen. They were analysed later on one occasion. Of the 881 participants, 717 delivered saliva samples. Several types of sampling error occurred, however. Principles were applied for the inclusion of some of these subjects since it was important to keep non-participation as low as possible:

- Twelve subjects failed to specify sampling time for one or several samples. Ten of these participants had specified sampling time on three of them but failed in one. The missing sample was assumed to correspond to the missing sampling time.

- Twenty-five subjects had failed to deliver at least one of the four samples. Four of them had missed the third sample, and in these cases the average of the saliva cortisol concentration was calculated from the second and fourth samples. This average replaced the missing value.

- Twenty-seven subjects had collected their four samples on two different consecutive days, for instance the fourth sample on day one and the remaining three on day two. All these were accepted.

After these inclusions and exclusions 685 subjects remained for analysis. Extreme outliers were identified. This resulted in the exclusion of seven subjects. Additional checking was made with regard to sampling time. Exclusions were made if sampling had occurred at grossly erroneous hours: For the first sample after 10.55 am, for the second after noon, for the third before 10.15 am or after 4 pm and for the fourth before 6.05 pm or after 3.00 am. Fifty participants were excluded for these reasons. Ninety-three of the remaining subjects were unemployed at the time of the study. They were also excluded. A final condition was that individuals who could not be categorized as exposed or unexposed on one of the demand and control dimensions were removed. Hence, the final study group consisted of 529 subjects (348 women and 181 men).

### Cortisol analysis

Cortisol levels in saliva were measured by the Spectria cortisol coated tube radioimmunoassay (RIA) kit, Orion Diagnostica, FI-02101 Spoo, Finland. All samples from each subject or group of subjects were analysed simultaneously in duplicate. The within- and between assay coefficient of variation never exceeded 5.0 and 10.0, respectively. A direct comparison of the cortisol analyses was made with Kirschbaum's laboratory in Düsseldorf. Thirty samples were analysed "blindly" in both laboratories and the results were compared. There was a very high correlation (0.98) but a slight difference in level – with systematically lower levels in the Stockholm laboratory. The difference was 12.5% with 95% confidence limits 1.5–22.3%.

### Explanatory variables

#### Psychological demands and decision latitude

Demand-control at work was assessed by means of the short Swedish version of the demand-control-support questionnaire [14-17]. There were five questions about psychological demands (for instance "Does your work require that you work fast?") and six questions about decision latitude (for instance "Can you influence what to do at work?" and "Is your work monotonous?"). All responses were graded from 1 to 4. Combinations of psychological demands and decision latitude were divided into four categories based upon medians. The "low strain" group was defined as those below the median for demands and above the median for decision latitude. The "passive" group consisted of those below the median for both demands and decision latitude and the "active" group of those above the median for both dimensions. The "high strain" group finally was defined as participants above the median for demands and below the median for decision latitude.

### Potential confounders

#### Medication

A physician examined the medications and coded them into three groups: Medications likely to have an effect on cortisol level (group I), medications which might have an effect on cortisol levels (group II) and medications with no suspected effects on cortisol levels (group III).

Substitution therapy with anabolic/androgenic steroids, estrogenes, gestagenes, gonadotropic releasing agents, hypophyseal hormones and pancreatic hormones as well as those on antihormonal therapy (anti estrogen and androgen) were regarded as belonging to group I. In the first step of the analyses of the effects of medications, these subjects (group I) were excluded (n = 54, 53 women and 1 man). Exclusion of subjects in group I had very small effects on mean levels and no effects on the significance levels in the comparisons between the mean saliva cortisol concentration in the different job-strain groups. In the following, those subjects in group I are included in the presentation of the results and in subsequent tables.

Group II medicines included a large number of medications that could potentially be of importance such as serotonin inhibitors as well as histamin 2 receptor inhibitors and similar medications for high production of acid in the stomach, glucocorticoids for local bowel treatment of gastrointestinal diseases, medication acting on the central nervous system for the treatment of obesity, beta receptor blockers, anticonception pills, ergot alkaloids and selective HT1 receptor inhibitors for the treatment of migraine, dopaminergic medication for the treatment of parkinsonism, anti-depressive medication (mono-amine reuptake inhibitors and selective serotonin uptake inhibitors as well as mono-amine inhibitors) and adrenergic medication for bronchial asthma and anti-histamine medications.

Confounding from medications in group II was tested by means of the introduction of this kind of medication as a confounder in statistical analysis. This was shown to be of very small importance. Accordingly we did not adjust for intake of these medications in the final analyses.

#### Other potential confounders

Full-time or part-time employment, severe life events [18], age, smoking, obesity, alcohol consumption measured by AUDIT (Alcohol Use Disorders Identification Test) [19], depression according to the Major Depression Inventory [20], and well-being, from the questionnaire in the second data collection were analysed as potential confounders of the relationship between job strain and saliva cortisol activity. However, all of them were shown to have very marginal and never any statistically significant confounding effects on relationships between job strain category and saliva cortisol. Therefore these analyses will not be presented.

### Statistical analysis

The participants provided repeated and mutually dependent cortisol samples and since our interest was on the comparison of the different demand-control categories with each other at each point in time we used a mixed model approach specifying a marginal means model. Specifically, we were interested in possible significant differences in saliva cortisol between demand control categories at half an hour after awakening and moreover in possible significant differences between demand control categories with regard to change in cortisol (CAR = Cortisol awakening response) between awakening and half an hour after awakening. The reason for these limited research questions was that we wanted to contribute to the understanding of how cortisol changes throughout the day may differ in different demand control categories as has been investigated by many others [2,4-8,21]. Thus, even when overall main effects of demand control category or the interaction effect, time*job strain was not significant, we still wanted to test specific relationships at these time points. Before using the mixed model procedure a check for normality of data was done which revealed that cortisol values were markedly skewed to the right. After a log transformation was performed the data were normally distributed. To be able to interpret the results an exponentiation was applied on the log-transformed estimates of cortisol for every demand/control group and point in time for men and women respectively. Hence, the remaining estimates are geometric means which could be interpreted as median values from a log-normal distribution of cortisol [22]. The time intervals between the different measurements varied somewhat between subjects in our study also after exclusion of subjects who had provided samples at times that deviated substantially from the specified scheme. These remaining differences had no effects on the results. A number of different variance-covariance patterns were fitted to the data. An unstructured pattern gave the best fit (lowest Akaike information criterion, AIC, than other patterns) [23] since it took into account different variances at specific time points and different covariances between each time point. This option was used in all analyses presented in this paper. *REML *(Restricted maximum likelihood) was used as the estimation method when trying different types of variance-covariance matrices. Maximum likelihood was used as the estimation method when building models. Separate models were built involving candidate confounding variables and demand-control category.

All the analyses were done in the SAS computer package version 9.1 using the MIXED procedure. The LSMEANS statement gives the possibility to estimate all median values for every group at every moment in time. The ESTIMATE statement gives the possibility to estimate the difference between groups at specific moments in time and estimates of differences in the mean changes between groups from one moment in time to another. In the REPEATED statement heterogeneity was specified as an option enabling different unstructured variance-covariance matrices for separate groups [24].

## Results

There were no differences between men and women with regard to when they produced their saliva samples. The second, third and fourth saliva sampling times were on average given for men and women respectively at 38 min (SD = 28 min) and 34 min (SD = 16 min) after awakening, 5h43 min (SD = 1h24 min) and 5h38 min (SD = 1h13 min) after awakening and 15h57 min (SD = 1h29 min) and 15h44 min (SD = 1h15 min) after awakening. When subdividing subjects into different demand-control categories the pattern did not change markedly neither for men or women.

Neither the main nor the interaction effects were significant for men or women. (see table 1)

Half an hour after awakening the high strain, active and passive female groups showed higher cortisol levels than the low strain group (p-values < 0.05). There was no significant difference between the four categories with regard to "slope" (difference between awakening and half an hour later or between half an hour later and lunch). For men no significant differences were found between active, passive and job strain categories (see table 2)

**Figure 1:**
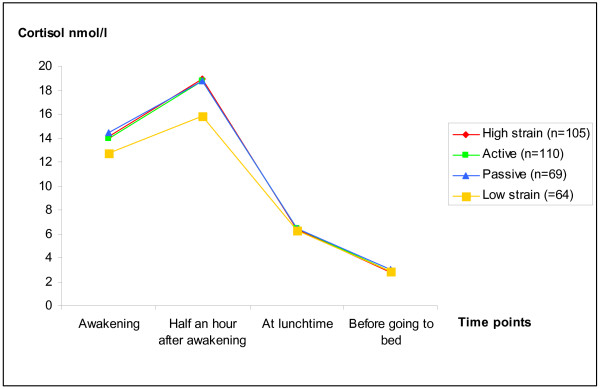
Median levels of cortisol in different job demand control categories among women at awakening, half an hour after awakening, at lunchtime and before going to bed.

The figure depicts the median cortisol values among women at the four time points for each demand control group. It shows a consistent difference between the low strain group and the other three groups both at awakening and at half an hour later. There are no differences between the active, passive and job strain groups (see figure 1).

## Discussion

Medication had surprisingly small effects on the median saliva concentration in the different groups. Accordingly, exclusion of subjects with medication that was judged to be particularly significant to cortisol excretion did not have any effects on the median saliva concentration of cortisol in the four categories. However, it could be that some of the more prevalent medications such as antidepressants restore the capacity to respond with elevated excretion of cortisol as a response to job stress. Similarly, analyses including medication as a possible confounder had very small effects on the results.

No significant demand-control*time point interactions were seen. There were, however, significant differences between the female groups in the separate analysis half an hour after awakening. Since there were no significant main or interaction effects in analysis of variance this finding should be interpreted with caution. Half an hour after awakening is probably the period when anticipation of a stressful day may exert strong effects on mobilisation of energy. Accordingly, despite the relative statistical weakness of these findings they are probably not random findings. It should also be pointed out, however, that first of all that there tends to be a difference between the low strain group and the others already at awakening and secondly that the rise in saliva cortisol concentration between awakening and half an hour later that has been discussed extensively in the literature did not differ between the demand control groups [2,21]. This could speak against the interpretation that we are dealing with an anticipation effect. One factor that could be of importance to this negative finding is the definition of "awakening". Whether subjects physically rise to their feet or not has been shown to be unimportant [25] but we may not have defined the point of "awakening" in a sufficiently standardised way in this study. Hence the true time lag between "awakening" and half an hour later may have varied considerably between subjects.

Another puzzling finding in this study was that the job strain, active and passive groups could not be differentiated from one another. We have no adequate explanation of this. According to the main hypothesis we should have found higher levels in the job strain group than in the active and the passive groups. All we can say is that there was no interaction between demand and control in the expected direction. Women reporting neither high demands nor low control had lower saliva cortisol than others half an hour after awakening.

There is a rapidly growing literature which relates both the demand control support model and the effort reward imbalance model to cortisol regulation. Steptoe et al have studied variations in saliva cortisol over the day in relation to overcommitment and the external part of the effort reward model [26]. That study which was based upon contrasting samples from the Whitehall II study showed that men who had high scores on the overcommitment scale had on average 22% higher saliva cortisol concentrations than men who had low scores. Comparisons between these groups also showed that the rise in saliva cortisol concentration from awakening to half an hour later was higher in overcommitted than in non-overcommitted men. No such findings were made in women. There is clearly a relationship between overcommitment and high demands. The external part of the effort reward score (a bad score indicates low reward for high effort) was not related to saliva cortisol levels neither in men nor in women in that study. Steptoe et al have also shown that teachers (men or women) with job strain have higher saliva cortisol levels at 800 and 830 in the morning than other teachers [7]. These associations were particularly strong in subjects who reported a high level of "anger out", tendency to react with openly expressed anger in stressful situations. A study of Japanese female health care workers showed higher urinary catecholamine output in those with self-reported job strain than in others. Saliva cortisol levels, on the other hand, were consistently lower in the job strain group than in the others [4].

Accordingly previous literature has shown that cortisol regulation is influenced by job stress. However the Japanese study shows that in some occupations early stages of physiological exhaustion leading to small differences between morning and evening cortisol excretion could be more prevalent in the job strain category than in the other categories [4]. The literature so far has accordingly produced inconsistent findings and our own study does not seem to provide any final answer. We only find weak support for the general demand control hypothesis. Women expecting neither high demands nor low control at work during their working day have a lower saliva cortisol level half an hour after awakening (perhaps when they have started to prepare themselves mentally for the upcoming conditions).

It is quite possible that there is a true difference between men and women. Kunz-Ebrecht et al showed that women seemed to react with more morning saliva cortisol elevation to the expectation of a stressful work day than men [21]. This could be one indication that women are more sensitive to work stress with regard to cortisol excretion than men but this has not been explored systematically in the literature. That the findings for men are non-significant in the present study might possibly also be due to a limited number of men.

The design of this study was based on selection of subjects with similar numbers from two groups. The groups consisted of those having low or high well-being, respectively, according to the WHO (Ten) Well-Being Index in the questionnaire in the second phase. It could be argued that a selection of subjects should have been done to secure similar numbers in the four different demand-control categories instead. Despite all, well-being was not related to cortisol and thus did not change the relation between job strain and cortisol. We also performed some analyses where the relations between job strain and cortisol were examined separately among those with high and low well-being. These analyses showed very similar patterns in the two well being groups. Low strain showed the lowest means in both groups while higher and comparable cortisol levels were found in the other three job strain categories in both groups.

## Conclusion

The circadian variation in saliva cortisol concentration was studied in a representative sample of actively working people in the greater Stockholm area. The study design allowed adjustment for a number of potential confounders such as mental state, medication, body mass index, smoking and alcohol consumption none of which had any significant impact on our findings. The results indicated that the saliva cortisol concentration was lower half an hour after awakening in women who reported neither high demands nor low decision latitude than in other working subjects. This association was only found in women and not in men, however. There was no difference between the three categories active, passive and high job strain. The findings only give weak support to the hypothesis that the demand control model is associated with saliva cortisol concentration.

## Competing interests

The author(s) declare that they have no competing interests.

## Authors' contributions

IL was responsible for the design of the PART-study, its questionnaire and data collection with regard to the data used for this paper. TT was responsible for the design of this particular study. MA was responsible for writing the manuscript with continuous input from all other authors. MA was also responsible for the statistical analyses and carried them out. BT analyzed the saliva samples. All authors contributed to the final version of the manuscript.

## Pre-publication history

The pre-publication history for this paper can be accessed here:



## Figures and Tables

**Table 1:** Main effects of job strain and interaction effects of job strain and time point on saliva cortisol in the total groups for men and women respectively.

			Main effect of job strain on cortisol	Interaction effect of time point*job strain on cortisol
	n	AIC-value ^1^	p-value	p-value

All women	348	2051,9	0,498	0,850
All men	181	1247,4	0,988	0,452

1. Akaike information criteria – values (AIC) are given as a measure of how well the model fits the data.

**Table 2:** Geometric mean values (GSE) of saliva cortisol in groups at different time points with p-values for the comparison with the low strain group and p-values for the comparison of the geometric mean change between different time points in the low strain group compared to the other groups. All women and men respectively.

	Job strain	Geometric mean values (GSE) in job strain groups at the different time points with p-values for the comparison between the low strain group				P-values (< 0.10) indicating significant differences in mean change between different time points Ref = Low strain		
		Awakening	Half an hour after awakening	At lunchtime	Before going to bed	Awakening – half an hour later	Half an hour after awakening – at lunchtime	At lunchtime – before going to bed

All women	High strain n = 105	14,13 (0,13)	18,90 (0,13) P = 0,016	6,38 (0,13)	2,81 (0,19)		P = 0,084	
	Active n = 110	14,00 (0,13)	18,77 (0,12) P = 0,020	6,44 (0,13)	2,82 (0,18)			
	Passive n = 69	14,45 (0,17)	18,74 (0,15) P = 0,036	6,42 (0,16)	2,98 (0,25)			
	Low strain n = 64	12,77 (0,17)	15,80 (0,16)	6,29 (0,16)	2,84 (0,26)		ref	

All men	High strain n = 37	12,82 (0,27)	17,36 (0,29)	7,62 (0,33)	3,21 (0,35)			
	Active n = 66	13,78 (0,26)	19,25 (0,19)	6,54 (0,21)	3,29 (0,31)			
	Passive n = 28	15,71 (0,27)	18,03 (0,30)	6,99 (0,29)	3,07 (0,36)			
	Low strain n = 50	14,95 (0,20)	18,11 (0,23)	7,22 (0,22)	2,78 (0,39)			
